# Study on the Preparation and Modification of a Novel Bio-Based Cardanol-Furfurylamine Oxazine Resin

**DOI:** 10.3390/polym17081084

**Published:** 2025-04-17

**Authors:** Jing Wang, Riwei Xu

**Affiliations:** 1 Basic Education School, Beijing Information Technology College, Beijing 100070, China; 2 Beijing Key Laboratory of Electrochemical Process and Technology for Materials, Beijing University of Chemical Technology, Beijing 100029, China

**Keywords:** cardanol, furfurylamine, benzoxazine, bio-based

## Abstract

In this study, a novel bio-based oxazine resin was synthesized through the reaction of naturally renewable materials: cardanol and furfurylamine. The molecular structure of the target product was confirmed via comprehensive characterization techniques, including Fourier-transform Infrared Spectroscopy (FT-IR), Gel Permeation Chromatography (GPC), Mass Spectrometry (MS), and Nuclear Magnetic Resonance (NMR). Differential Scanning Calorimetry (DSC) revealed that the curing process of cardanol-furfurylamine oxazine (CFZ) exhibited three exothermic peaks (~140, ~240, ~270 °C), which not only helped to optimize the processing conditions but also effectively enhanced the material properties. In the modification experiments, CFZ had been blended and cured with benzoxazine (BZ) at the mass ratios of 2:98, 5:95, 10:90, 20:80, and 40:60. Dynamic Mechanical Thermal Analysis (DMTA) further showed an elevated Loss Factor (tan δ) peak of CFZ-BZ resin, suggesting significantly enhanced toughness. Notably, when the content of the CFZ resin in the composite reached only 5%, the storage modulus achieved its maximum value, highlighting that minimal addition of CFZ resin can optimize the rigidity of the composite, which would drastically reduce material costs and simplify the process. Impact strength testing demonstrated that the impact resistance of CFZ-BZ resin was 6.42 times higher than that of pristine BZ. By integrating renewable materials with rational molecular design, this novel oxazine resin synergistically combines high-temperature resistance, superior toughness, and efficient modification at low loading, positioning it as a promising candidate to replace conventional petroleum-based resins in aerospace, renewable energy, and electronic packaging applications.

## 1. Introduction

Benzoxazine resin is a high-performance thermoset polymer. Since its systematic research and promotion by Japanese scholar H. Ishida and others in the 1990s, it has gradually become a research hotspot in the field of polymer materials. Its molecular structure is centered on the benzo-oxazine ring and a highly crosslinked three-dimensional network structure is formed during the ring-opening polymerization [1]. Compared to traditional phenolic resins, benzoxazine resins offer superior toughness and can be molded into specimens without the need for additional modifiers. Their curing process generates no by-products, contributing to a favorable environmental profile. Furthermore, their minimal volumetric shrinkage upon curing reduces the likelihood of phase separation between the matrix resin and dispersed phase, which could otherwise compromise the mechanical properties of composite materials.

Recognizing the distinctive properties of benzoxazine resins, researchers have conducted extensive investigations into their applications. Cong Peng et al. [2] prepared a phosphorus-containing benzoxazine monomer, which was chemically bonded with 9,10-dihydro-9-10-oxa-10-oxide to produce an epoxy/benzoxazine copolymer. Kan Zhang et al. [3] produced an interesting benzoxazine resin via phenol (naringenin) and amine (furfurylamine), which had a long shelf life. Seishi Ohashi et al. [4] synthesized the first benzoxazine structure containing a cyanate group. Vidhukrishnan et al. [5] synthesized a new benzoxazines containing polyformaldehyde from vanillin. Shuai Zhang [6] prepared a kind of polybenzoxazine resins with high electromagnetic interference shielding effectiveness. Xiaotian Fan et al. [7] synthesized a new fluoropyridine-based benzoxazine resin with excellent flame retardant properties and a large water contact angle. Hariharan [8] adopted a novel imidazole-core-based bisphenol and prepared a benzoxazine resin with higher char yields. Siqi Huo [9] and Chunxia Zhao [10] prepared a benzoxazine resin with excellent flame-retardant properties. Su Tao et al. [10] enhanced the toughness and strength of benzoxazine monomers by incorporating polysulfide rubber during their synthesis, followed by co-curing with epoxy resin.

Because of the diverse structural characteristics and unique functions, natural renewable materials have become ideal raw materials to prepare high-performance benzoxazine resins. Some researchers have begun to synthesize various new benzoxazine monomers from nature, or from agricultural waste or environmental industrial by-products [11,12]. Dr. Cody et al. [13] were inspired by biological design and adopted bioavailable phenolic compounds to introduce the catechol functional group into the benzoxazine monomers, generating a derivative of polybenzoxazine with high shear strength.

Cardanol, a natural phenolic compound extracted from cashew nutshell oil, contains a high proportion of reactive monophenols and a small fraction of diphenols. Its benzene ring structure imparts thermal stability, while the polar hydroxyl groups enhance interfacial wettability and reactivity. The meta-positioned long-chain alkyl substituents (C^15^ unsaturated hydrocarbon chains) reduce the crosslinking density of the resin, thereby improving toughness and impact resistance. Additionally, these long-chain alkyl structures contribute to superior hydrophobicity, low permeability, and air-drying properties.

This study aims to develop a novel bio-based benzoxazine resin derived from cardanol and furfurylamine via molecular design. The combination of the rigid benzene ring and flexible long-chain structure of cardanol ensures an optimal balance between rigidity and flexibility, while the crosslinking between the amine groups of furfurylamine and the hydroxyl groups of cardanol forms a dense network, enhancing the strength and toughness of the benzoxazine resin. The molecular structure of the synthesized resin will be characterized via Infrared Spectroscopy (IR), Gel Permeation Chromatography (GPC), Mass Spectrometry (MS), and Nuclear Magnetic Resonance (NMR), along with an evaluation of its performance. Furthermore, the study will explore the modification of benzoxazine resins using this novel resin and optimize the composite materials that have the best storage modulus.

The raw materials for this novel benzoxazine resin are entirely derived from natural and renewable resources. Owing to its bio-based origin, flexibility, and multifunctionality, it is expected to open new avenues in aerospace, renewable energy, and electronic packaging applications. Its key value lies in integrating the sustainability advantages of natural compounds with the high performance of traditional benzoxazines, positioning it as a promising alternative to petroleum-based resins in the future.

## 2. Materials and Methods

### 2.1. Material Preparation

#### 2.1.1. Preparation of Cardanol-Furfurylamine Oxazine (CFZ) Monomer

**Scheme 1:**
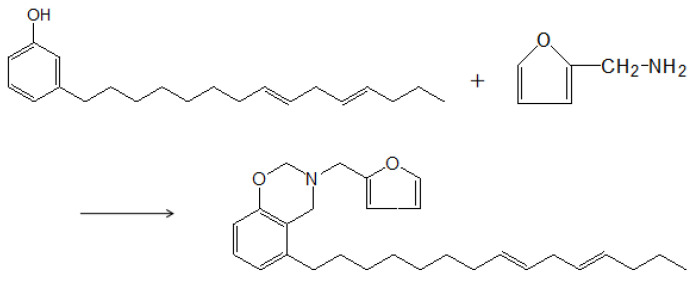
The reaction equation of the CFZ monomer.

In a three-neck round-bottom flask (500 mL) equipped with a condenser and a mechanical stirrer, 200 mL of toluene (Beijing Beihua Fine Chemicals Co., Ltd., Beijing, China) was first added as the solvent. Subsequently, 75.1 g of cardanol (0.25 mL, Hdsg Beijing Technology Co., Ltd., Beijing, China) and 24.3 g of furfurylamine (0.25 mol, Beijing Institute of Chemical Reagents Co., Ltd., Beijing, China) were introduced sequentially. Under vigorous stirring in an ice-water bath, 30 g of paraformaldehyde (0.5 mol, Tianjin Institute of Chemical Reagents, Tianjin, China) was added slowly. The reaction mixture was then stirred intensely at room temperature to ensure a uniform mixture before being heated in an oil bath at 95 °C for 2 h. After completion of the reaction, 1 mol/L sodium hydroxide (Beijing Wuzhou Century Red Star Chemicals Ltd., Beijing, China) solution was added and stirred thoroughly. The mixture was subsequently transferred to a separatory funnel to isolate the product, which was further washed with sodium hydroxide solution and then rinsed with deionized water until neutral. Finally, the product was poured into a porcelain dish and dried under vacuum at 60 °C for 24 h, yielding an off-white solid. The reaction equation can be seen as Scheme 1.

#### 2.1.2. Preparation of Benzoxazine (BZ) Monomer

**Scheme 2:**
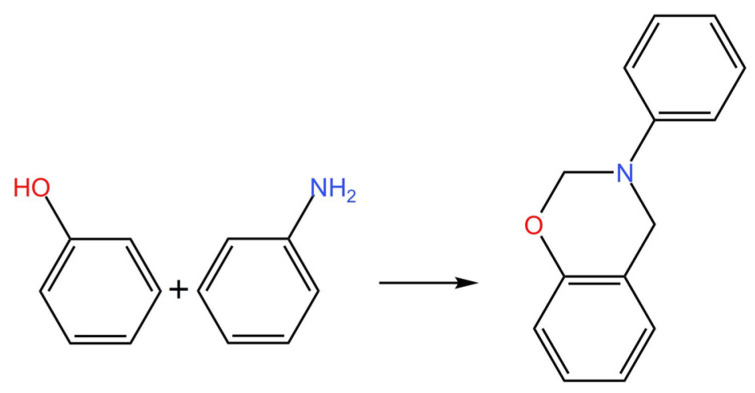
The reaction equation of the BZ monomer.

In a 500 mL three-necked flask, 200 mL of toluene was first added as the solvent. Subsequently, 23.5 g of phenol (0.25 mol) and 23.28 g of aniline (0.25 mol) were sequentially introduced. Under an ice-water bath with vigorous stirring, 30 g of paraformaldehyde (0.5 mol) was slowly added. The reaction system was then warmed to room temperature and continuously stirred to ensure the thorough mixing of the reactants. Afterward, the reaction mixture was heated in an oil bath at 95 °C for 2 h. Upon completion of the reaction, an aqueous sodium hydroxide solution (1 mol/L) was added and stirred for homogenization. The resulting mixture was transferred to a separatory funnel for phase separation. Subsequently, the product was further washed with sodium hydroxide solution and rinsed with deionized water until neutral. Finally, the product was transferred to a porcelain dish and vacuum-dried at 60 °C for 24 h, yielding a light brown solid. The reaction equation can be seen as Scheme 2.

#### 2.1.3. Preparation of CFZ-BZ Resin

A new composite material was synthesized through the blending and curing of the CFZ monomer and BZ monomer. Using the solution blending method, the CFZ monomer and BZ monomer were dissolved in a specified amount of tetrahydrofuran (THF, Beijing Beihua Fine Chemicals Co., Ltd., Beijing, China) at predetermined mass ratios of 2:98, 5:95, 10:90, 20:80, and 40:60. The solutions were stirred for 5 h, followed by vacuum evaporation to remove the solvent. A measured amount of the resulting blend was then transferred into a mold and cured under specific process conditions (heated at 120 °C for 2 h, 140 °C for 2 h, 160 °C for 2 h, 180 °C for 3 h, 200 °C for 2 h) in a vacuum oven to achieve a CFZ-BZ resin.

### 2.2. Test and Characterization

To confirm the structure and composition of the synthesized product, a Nicolet-60SXB Fourier Transform Infrared (FTIR) spectrometer (Thermo Nicolet Inc., Madison, WI, USA) was employed to analyze the functional groups in the molecular structure of CFZ. The sample was prepared using the KBr pellet method and the measurement wave-number range was set from 400 to 4000 cm^−1^.

The molecular weight and its distribution were determined via a Waters GPC 515-2410 system (Waters Inc., Milford, MA, USA), in which Styragel HT3, HT5, and HT6E columns were used in series at a controlled temperature of 30 °C, with THF as the mobile phase (2 mg/mL; 1 mL/min).

CFZ’s composition was further validated via a Waters Quattro Premier XE quadrupole tandem mass spectrometer (MS; Waters Inc., Milford, MA, USA), at a cone voltage of 30 V.

The molecular structure was determined using a Bruker Avance 600 MHz Nuclear Magnetic Resonance (^1^H-NMR) spectrometer (Bruker Inc., Karlsruhe, Germany). The sample was dissolved in CDCl_3_, with Si(CH_3_)_4_ as an internal standard, and the spectrum was drawn at room temperature.

To further evaluate the comprehensive performance of CFZ, a PerkinElmer TGA Pyris 1 thermal analyzer (PerkinElmer Inc., Waltham, MA, USA) was employed to analyze the thermal stability of the product. The heating rate was set to 10 °C/min (from room temperature to 350 °C), using Al_2_O_3_ as the reference material.

The mechanical properties of CFZ-BZ resin were examined via a Rheometric Scientific DMTA V dynamic mechanical thermal analyzer (Rheometric Scientific, Inc., Piscataway, NJ, USA), with sample dimensions of 25 × 6.5 × 1.5 mm^3^. The heating rate was set to 5 °C/min (from room temperature to 250 °C) and the testing frequency was set at 1 Hz under a single cantilever bending mode.

The impact resistance of CFZ-BZ resin was assessed via a simply supported beam pendulum impact tester (P/N 6957.000, Dongguan Municipal People’s Instrument and Equipment Co., Ltd., Dongguan, China). The test was conducted following the GB/T 2571-1995 standard [14], with specimen dimensions of 80 × 10 × 4 mm^3^, and ten specimens were prepared for each mass ratio, while the span length was set to 70 mm.

## 3. Results and Discussion

### 3.1. Optimization of Synthetic Conditions

According to the synthesis process described in Section 2.1.1, different solvents, including n-hexane (Beijing Beihua Fine Chemicals Co., Ltd., Beijing, China), toluene, and nitromethane (Beijing Beihua Fine Chemicals Co., Ltd., Beijing, China), were selected to evaluate the yield of CFZ monomer, as shown in Table 1. The results indicate that no target product was obtained when the solvent had either a very low or a high dielectric constant. This phenomenon can be attributed to the fact that the formation of the oxazine ring shall proceed via a Mannich-type condensation reaction [15], wherein highly polar solvents (i.e., those with higher dielectric constants) adversely affect the stability of intermediates, the efficiency of dehydration, and the activity of formaldehyde, thereby hindering product formation [16]. n-Hexane has an extremely low dielectric constant and it is almost nonpolar, resulting in poor solubility. Consequently, toluene was selected as the solvent in this study.

Under other identical reaction conditions, different aldehyde sources were employed while a constant molar quantity was maintained. Specifically, paraformaldehyde, trioxane, and an aqueous formaldehyde solution were selected for investigation. As shown in Table 2, the highest yield of CFZ was obtained when paraformaldehyde was used. This can be attributed to the fact that paraformaldehyde undergoes gradual depolymerization under heating conditions, releasing monomeric formaldehyde in a controlled manner. The regulated formaldehyde release rate aligns well with the condensation and cyclization reactions, while the anhydrous system prevents hydrolysis and interference from acidic conditions [17]. However, trioxane requires a higher temperature for depolymerization, which may result in insufficient formaldehyde supply. Therefore, paraformaldehyde was chosen as the preferred reactant.

**Figure 1:**
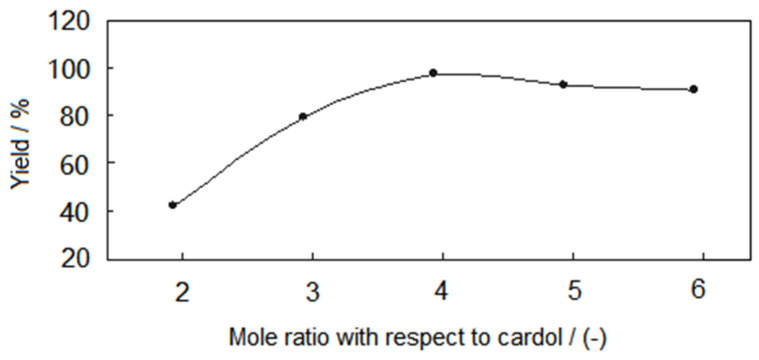
Trend of yield with varying paraformaldehyde amounts in the reaction.

Upon determining the use of toluene as the solvent, the amount of paraformaldehyde was varied to achieve the molar ratios of 2:1:1 (theoretical), 3:1:1, 4:1:1, 5:1:1, and 6:1:1 with respect to cardanol and furfurylamine. From Table 3 and Figure 1, it can be seen that the yield of CFZ increased with the addition of paraformaldehyde, as formaldehyde serves as a key crosslinking agent in the reaction. Within an appropriate range, its increased presence promotes the condensation reaction between hydroxyl (–OH) and amine (–NH_2_) groups, thereby enhancing the degree of crosslinking and improving the overall yield. However, excessive formaldehyde leads to side reactions, where the –NH_2_ group of furfurylamine undergoes multiple-step reactions with excess formaldehyde, forming methylamine derivatives or other by-products, ultimately reducing the yield [18]. Consequently, the highest yield was achieved at a molar ratio of 4:1:1.

### 3.2. Characterization of CFZ

To further verify whether the theoretical molecular design aligns with the actual structure of CFZ, a series of tests and characterizations were performed on the product.

**Figure 2:**
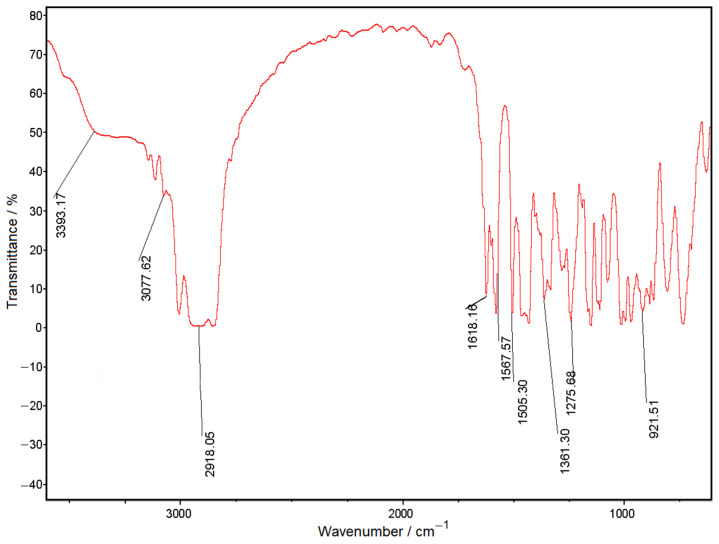
The FTIR spectrum of CFZ.

Figure 2 presents the FTIR spectrum of CFZ, where a characteristic absorption peak of oxazine ring is distinctly observed at 921 cm^−1^. Additionally, the absence of an –OH stretching vibration peak around 3393 cm^−1^ indicates that the phenolic hydroxyl groups have fully used up, confirming the successful formation of the oxazine ring [19]. The absorption peak at 1275 cm^−1^ corresponds to the methylene rocking vibration within the oxazine ring, further supporting its structural integrity. Moreover, the peak at 3077 cm^−1^ is attributed to the asymmetric C–H stretching vibration of the C=C bond in the allyl group, while the absorption at 1618 cm^−1^ corresponds to the C=C stretching vibration of the allyl group, suggesting that the allyl structure in the cardanol side chain remains unreacted. The characteristic skeletal vibration peaks of the benzene ring (C=C stretching) appear at 1567 and 1505 cm^−1^, confirming the retention of the aromatic structure. Additionally, the peak at 2918 cm^−1^ corresponds to the asymmetric stretching vibration of –CH_3_, whereas the absorption at 1361 cm^−1^ is assigned to the symmetric deformation vibration of the methyl group, verifying the presence of the methyl group on the cardanol side chain. In summary, the target product was successfully synthesized with high selectivity, as only the phenolic hydroxyl groups participated in the condensation reaction, while the side-chain structure remained intact.

**Figure 3:**
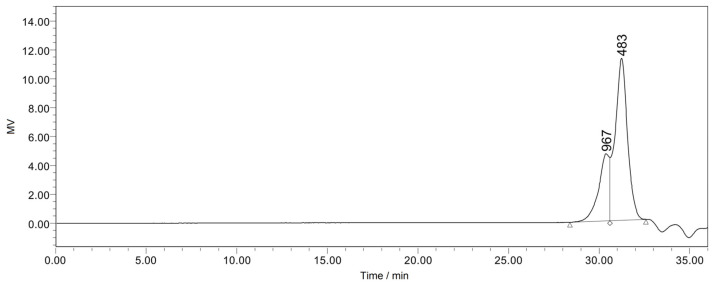
The GPC chromatogram of CFZ (△: mark the starting and ending points of the baselines; ◊: mark the boundary between two peaks).

The molecular weight and distribution of the product were analyzed via a GPC and MS test. Figure 3 presents the GPC chromatogram of CFZ, which reveals a relatively narrow molecular weight distribution. The main peak is concentrated at 483, while a minor peak appears at 967, suggesting the possible formation of both monomers and dimers [20].

**Figure 4:**
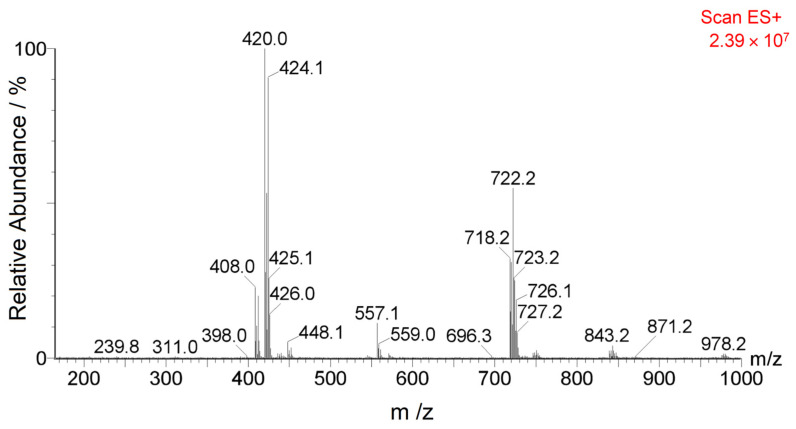
The MS of CFZ.

**Scheme 3:**
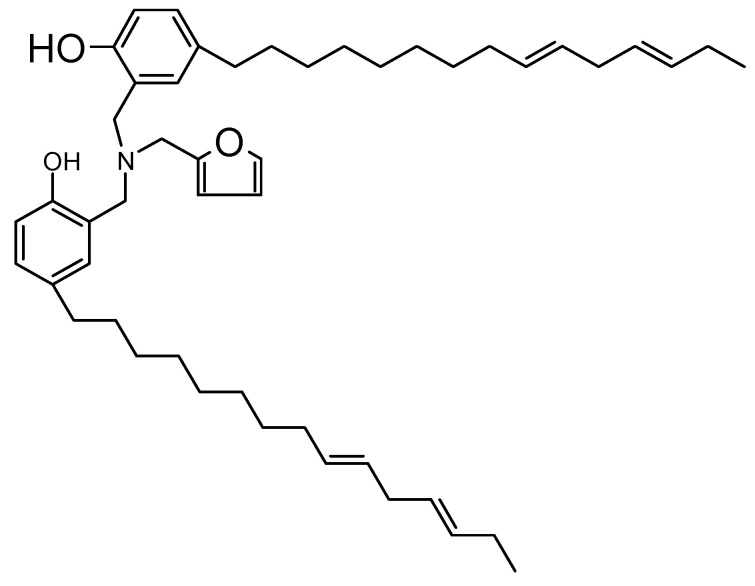
The dimer produced during the reaction process.

However, GPC exhibits certain limitations in determining small molecules accurately, necessitating further confirmation of the product composition via MS (Figure 4). The calculated molecular weight of CFZ is 421.3, which is consistent with the measured result in the spectrum. The peak corresponding to 424 represents a long-branched-chain isomer of CFZ, which features a saturated long carbon chain structure. The molecular formula shown in Scheme 3 corresponds to the dimer formed during the reaction, with a theoretical molecular weight of 722, which matches the peak observed in the MS. Additionally, the peaks at 726 and 727 shall be attributed to the isomers of the dimer containing a saturated long carbon chain structure [20].

**Figure 5:**
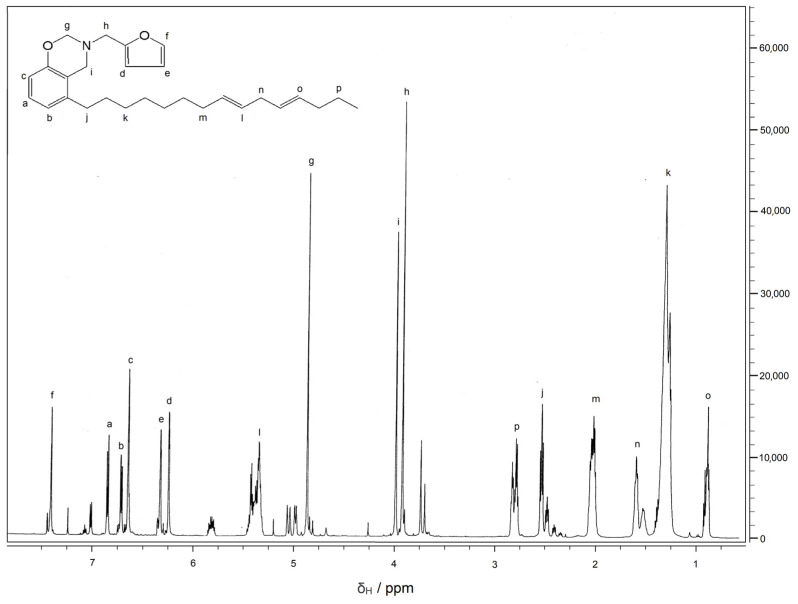
^1^H-NMR spectrum of CFZ.

Figure 5 presents the ^1^H NMR spectrum of CFZ. It can be observed that the chemical shift at δ = 6.663–6.872 corresponds to protons on the benzene ring, indicating the integrity of the aromatic structure. The multiple peaks in the range of 5.347–5.445 suggest the presence of a C=C double bond in the molecule. The signal at δ = 4.882 corresponds to the -O-CH_2_-N- proton, while the peak at δ = 3.937 is assigned to the -Ar-CH_2_-N- proton, further supporting the existence of the oxazine structure in the molecule. The peak at δ = 2.549 corresponds to the -Ar-CH_2_-C proton, indicating that the aromatic ring is connected to a methylene group, which is further linked to a carbon chain, consistent with the expected molecular structure. The signal at δ = 1.334 is attributed to the protons of the -CH_2_- long alkyl chain, while the multiple peaks at δ = 0.901 correspond to terminal methyl -CH_3_ groups. Due to the possible presence of constitutional isomers (e.g., branched chains or cis-trans isomers) in the long alkyl chain of cardanol, protons in different conformations or chemical environments exhibit varying chemical shifts, leading to the appearance of additional signals [21].

In the figure, the peak area ratio is observed as: a:b:c:d:e:f:g:h:i:j:k:l:m:n:o:p = 0.90:0.96:1.17:1.00:1.03:0.99:2.01:2.04:2.00:2.02:9.73:4.16:4.01:1.96:1.68:2.86, while the theoretical ratio is: a:b:c:d:e:f:g:h:i:j:k:l:m:n:o:p = 1:1:1:1:1:1:2:2:2:2:10:4:4:2:2:3. (The detailed data can be seen in Appendix A).

From the above data, it can be observed that the chemical shifts of hydrogen atoms at different positions in the product allow for the assignment of each proton in the molecule. Moreover, the experimental peak area ratios are in close agreement with the theoretical values, further confirming the expected proton distribution. Based on this analysis, it can be inferred that the final reaction product corresponds to the designed target compound [22].

**Figure 6:**
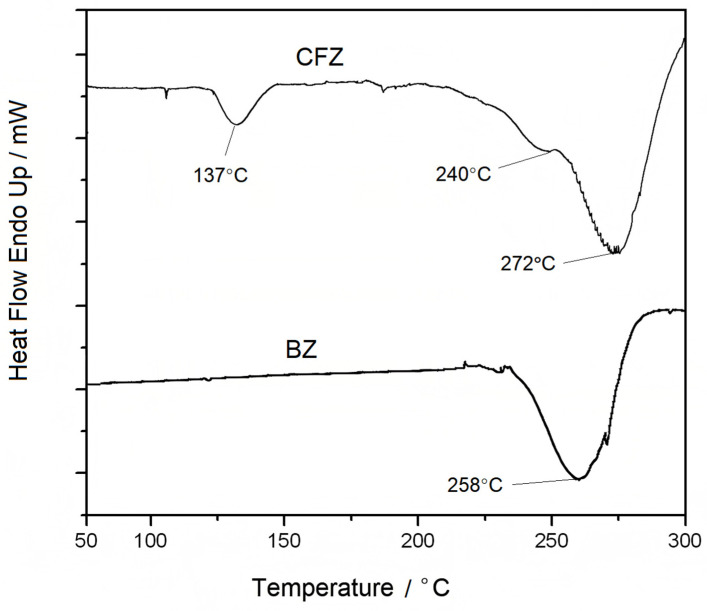
DSC curing curve of CFZ and BZ.

From the DSC curing curve of CFZ (Figure 6), the three exothermic peaks suggest that the curing process of the CFZ resin involves multiple reaction stages, each corresponding to the curing of different chemical bonds, which indicates that the system undergoes a multi-step curing process. The first exothermic peak appears at approximately 140 °C, corresponding to the free radical polymerization of the long-chain double bonds. The second exothermic peak appears at approximately 240 °C, indicating that this stage corresponds to the crosslinking reaction between the double bonds on the furan ring and those on the long alkyl chain. The third exothermic peak, observed around 270 °C, corresponds to the ring-opening polymerization of the oxazine ring, which is a characteristic curing behavior of oxazine-based systems [23]. Figure 6 also shows the DSC curve of BZ, where a prominent exothermic peak can be observed around 258 °C, indicating the occurrence of ring-opening polymerization that forms a highly crosslinked, heat-resistant thermoset network [24]. In contrast, CFZ offers more advantages in terms of controllable curing processes and the design of flexible materials.

### 3.3. Performance Testing of CFZ-BZ Resin

**Figure 7:**
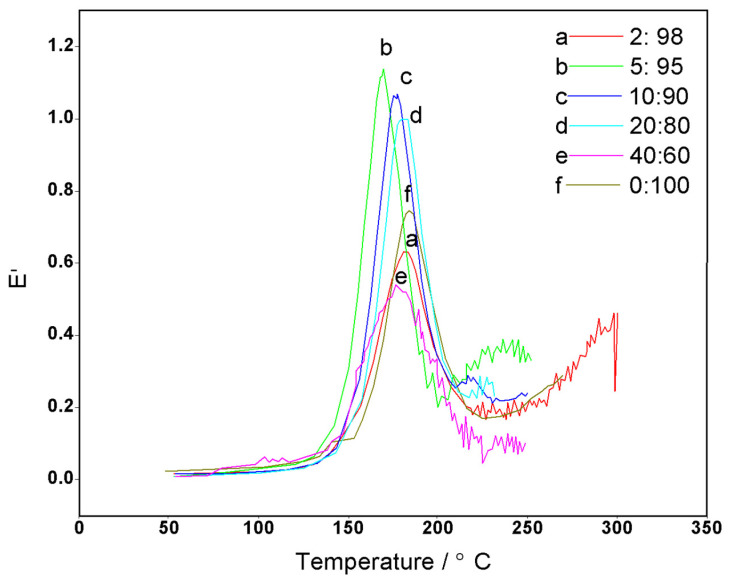
Graph of tan δ versus temperature for BZ resins with different CFZ contents.

**Figure 8:**
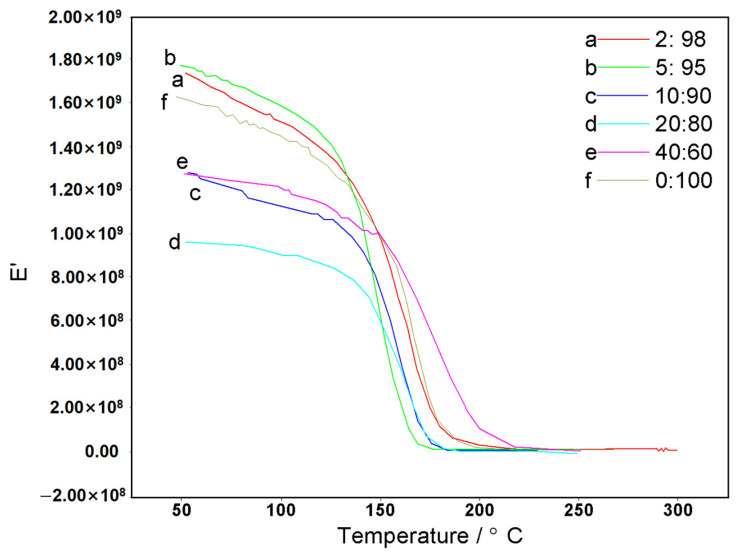
Graph of the storage modulus versus temperature for BZ resins with different CFZ contents.

The obtained CFZ-BZ-modified specimens were subjected to DMA testing and the results are shown in Figure 7 below. It can be observed that the addition of CFZ to BZ resin leads to an increase in the tan δ peak value, indicating the enhanced molecular chain segment mobility within the system. This may be attributed to the incorporation of the more flexible long alkyl chain in the CFZ structure, which reduces intermolecular hydrogen bonding density and facilitates chain segment motion. However, the temperature range of the peak narrows and the peak shifts toward a lower temperature, suggesting that the distribution of the glass transition temperature (Tg) becomes narrower and the cured network structure is more uniform. Meanwhile, the increase in the proportion of flexible chain segments likely reduces the overall crosslinking density of the system. Figure 8 demonstrates that when the CFZ content reaches 5%, the storage modulus increases significantly, indicating that the system achieves the optimal curing state and network reinforcement at this composition. At this point, the material simultaneously maintains a high storage modulus while retaining a certain level of flexibility. However, the increase of CFZ content may lead to further reduction in crosslinking density and result in a decrease in the storage modulus [25].

**Figure 9:**
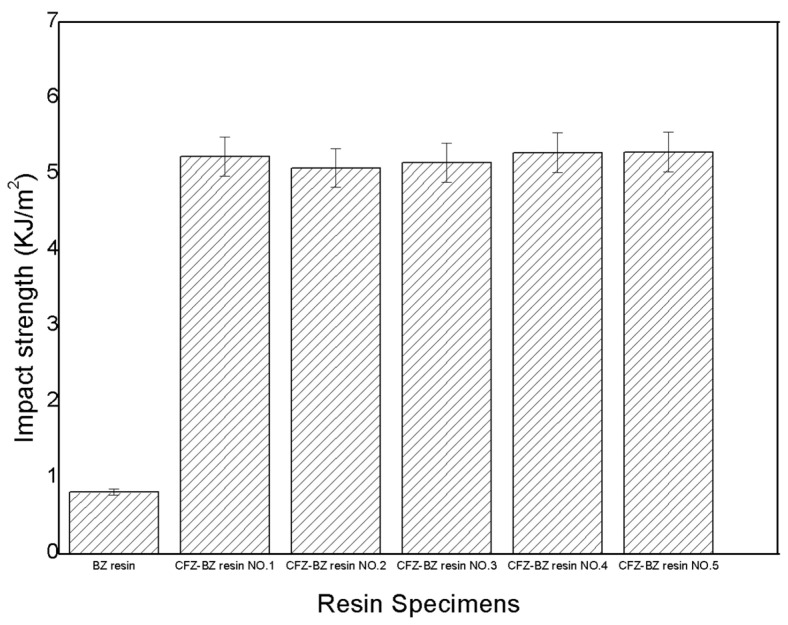
The impact strength comparison of resin specimens.

Based on the aforementioned study, CFZ-BZ resin specimens with a CFZ content of 5% were selected for the impact strength tests. The results are presented in the Table 4 and Figure 9 below, showing that the impact strength of the blended resin increased by an average of 6.42 times, compared to the unmodified BZ resin. This indicates a significant enhancement in the impact toughness of the system, which may be attributed to the long alkyl chains in the CFZ structure with flexible segments. This incorporation might reduce the cohesive energy of benzoxazine, increases molecular chain mobility, and allows the material to undergo a certain degree of plastic deformation under external forces rather than fracturing immediately [26]. Furthermore, the introduction of CFZ adjusts the crosslinking density, leads to a more uniform cured network, and reduces localized stress concentrations, thereby improving the fracture toughness of the system.

## 4. Conclusions

In this study, a novel bio-based resin with excellent performance was synthesized from cardanol and furfurylamine via molecular structure design. The structure of CFZ was characterized and confirmed via FTIR, GPC, MS, and ^1^H NMR tests, demonstrating consistency with the designed structure and verifying it as the target product. DSC analysis revealed a more complex curing process for this oxazine resin, characterized by three exothermic peaks: polymerization of long-chain double bonds (~140 °C), crosslinking reaction (~240 °C), and oxazine ring-opening polymerization (~270 °C), which indicates that the resin exhibits not only high thermal stability but also enhanced flexibility. A series of CFZ/BZ resins were cured with CFZ contents at 2:98, 5:95, 10:90, 20:80, and 40:60, followed by DMA test. The results showed that CFZ effectively enhanced the tan δ value of the blend resin. Notably, when the CFZ content reached 5%, the storage modulus of CFZ/BZ resin significantly increased, suggesting that at this composition, the curing degree and network structure achieved an optimal reinforcement state, allowing the material to maintain both high storage modulus and a certain level of flexibility. Impact strength testing of the cured CFZ/BZ resin further demonstrated that its impact resistance was 6.42 times higher than that of pure BZ resin, indicating a substantial improvement in toughness. The development of this novel bio-based oxazine resin not only supports environmental sustainability but also offers exceptional thermal resistance, flexibility, and rigidity, making it a promising candidate for high-performance composite materials with significant potential as a substitute for conventional resins.

## Figures and Tables

**Table 1:** The relationship between solvent and yield.

Solvent	Dielectric Constant (20 °C)	Product Yield	Product Status
n-Hexan	1.89	————	————
Toluene	2.24	58.56%	Yellow oily viscous liquid
Nitromethane	4.33	————	————

————: There had been no product produced.

**Table 2:** The relationship between aldehydes and yield.

Aldehydes	Theoretical Output/g	Actual Output/g	Yield
Paraformaldehyde	41.1	17.04	41.46%
Trioxane	41.1	————	————
Aqueous formaldehyde solution	41.1	10.04	24.43%

————: There had been no product produced.

**Table 3:** The relationship between yield and aldehyde content.

Mole Ratio	Theoretical Output/g	Actual Output/g	Yield
2:1:1	41.1	17.04	41.46%
3:1:1	41.1	32.54	79.17%
4:1:1	41.1	39.78	96.79%
5:1:1	41.1	37.64	91.58%
6:1:1	41.1	37.12	90.32%

**Table 4:** The impact strength of BZ resin and CFZ-BZ resin.

Name of Resins	Impact Strength KJ/m^2^	Impact Strength Ratio (CFZ-BZ/BZ)	Average Ratio
BZ resin	0.81	——	——
CFZ-BZ resin NO.1	5.23	6.45	6.42
CFZ-BZ resin NO.2	5.08	6.27	
CFZ-BZ resin NO.3	5.15	6.35	
CFZ-BZ resin NO.4	5.28	6.51	
CFZ-BZ resin NO.5	5.29	6.53	

**Figure A1:**
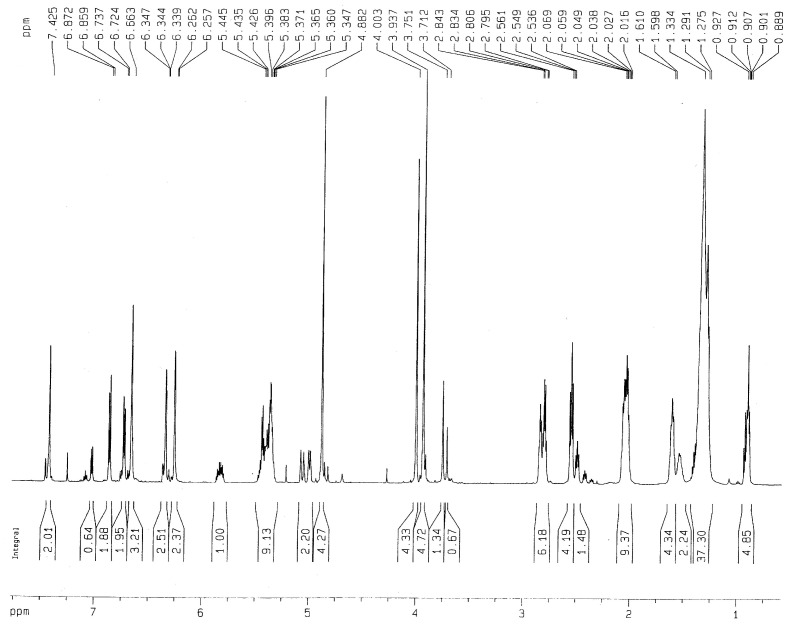
^1^H NMR spectrum with chemical shifts and integral values of CFZ.

## Data Availability

The data presented in this study are available on request from the corresponding author due to privacy.

## Abbreviations

The following abbreviations are used in this manuscript:

CFZ	Cardanol-furfurylamine oxazine
BZ	Benzoxazine
FT-IR	Fourier-transform Infrared Spectroscopy
GPC	Gel Permeation Chromatography
MS	Mass Spectrometry
NMR	Nuclear Magnetic Resonance
DSC	Differential Scanning Calorimetry
DMTA	Dynamic Mechanical Thermal Analysis

## Appendix A

For more detailed chemical shifts, integral values, and splitting patterns, please refer to the Figure A1 below.
